# Like iron in the blood of the people: the requirement for heme trafficking in iron metabolism

**DOI:** 10.3389/fphar.2014.00126

**Published:** 2014-06-04

**Authors:** Tamara Korolnek, Iqbal Hamza

**Affiliations:** ^1^Department of Animal & Avian Sciences, University of Maryland, College ParkMD, USA; ^2^Department of Cell Biology & Molecular Genetics, University of Maryland, College ParkMD, USA

**Keywords:** heme, iron, transport, hematology, porphyrins, anemia, erythropoiesis, nutrition disorders

## Abstract

Heme is an iron-containing porphyrin ring that serves as a prosthetic group in proteins that function in diverse metabolic pathways. Heme is also a major source of bioavailable iron in the human diet. While the synthesis of heme has been well-characterized, the pathways for heme trafficking remain poorly understood. It is likely that heme transport across membranes is highly regulated, as free heme is toxic to cells. This review outlines the requirement for heme delivery to various subcellular compartments as well as possible mechanisms for the mobilization of heme to these compartments. We also discuss how these trafficking pathways might function during physiological events involving inter- and intra-cellular mobilization of heme, including erythropoiesis, erythrophagocytosis, heme absorption in the gut, as well as heme transport pathways supporting embryonic development. Lastly, we aim to question the current dogma that heme, *in toto*, is not mobilized from one cell or tissue to another, outlining the evidence for these pathways and drawing parallels to other well-accepted paradigms for copper, iron, and cholesterol homeostasis.

## INTRODUCTION

Heme is an iron-containing porphyrin that functions as a cofactor in a wide array of cellular processes. Heme is also a major source of bioavailable iron in the human diet. By the end of the 20th century, the pathways for heme biosynthesis had been well elucidated and the structures of many heme-containing proteins have been solved. Indeed, Max Perutz and John Kendrew were awarded the Nobel Prize in chemistry in 1962 for their crystal structures of hemoglobin and myoglobin (Green et al., 1954; Kendrew et al., 1958). While the synthesis of heme has been well-characterized, the pathways for inter- and intra-cellular heme transport remain poorly understood. This gap in our knowledge is largely due to the inability to uncouple the processes of heme biosynthesis and heme transport, as well as heme’s ability to promiscuously bind to proteins. By contrast, great strides have been made in the fields of iron and copper trafficking, which have not suffered similar setbacks. Transport pathways for non-heme iron have been studied for decades and, all the while, knowledge of heme-iron trafficking has languished. The title of this review includes a verse from the poem “Kids Who Die” by the American poet Langston Hughes raising the possibility that heme too moves *like iron in the blood of the people*.

This review aims to (a) outline the biological requirement for heme trafficking pathways in eukaryotes, including the inability of heme to efficiently traverse lipid membranes and the presence of hemoproteins in almost all subcellular compartments, (b) summarize how these putative pathways may participate in various biological processes, including the synthesis and recycling of red blood cells (RBCs), intestinal absorption of dietary heme, embryogenesis, and (c) summarize the evidence for inter-cellular and inter-tissue transport of heme.

## MOVEMENT OF HEME ACROSS MEMBRANES

The terminal step of heme biosynthesis, which occurs in the mitochondrial matrix, is the insertion of iron into protoporphyrin IX. As will be discussed in depth in this article, heme is then targeted to both soluble and membrane-bound hemoproteins. Heme must be able to cross membranes, yet also be delivered to soluble proteins in the cytosol. Heme’s amphipathic nature means that it is able to function in both milieus, but not without the assistance of partners. Due to the hydrophobicity of the porphyrin ring, heme is not readily soluble in aqueous solutions. When heme is at neutral pH and is in the ferrous state it has no net charge, as the negative charges of the propionate groups balance the positive charges of the chelated iron. While this form of heme readily inserts into and diffuses through lipid bilayers, the hydrophilic propionate head groups hinder the flipping of heme from one leaflet to another (Cannon et al., 1984; Rose et al., 1985; Light and Olson, 1990). Heme exit from the membrane is inefficient without the presence of soluble heme-binding proteins (HBPs; Yoda and Israels, 1972).

In addition to the difficulties posed by heme’s dual chemical nature, heme is highly toxic due to its peroxidase activity and capacity to generate reactive oxygen species combined with its ability to intercalate into membranes and bind many proteins non-specifically. The toxic effects of heme have been reviewed extensively in mammalian cells and other systems (Vincent, 1989; Jeney et al., 2002; Oliveira et al., 2002; Kumar and Bandyopadhyay, 2005; Chow et al., 2008; Larsen et al., 2012). Thus, even though heme is able to diffuse into membranes and be extracted by HBPs, it is likely that this process would be regulated, as peroxidation of membrane lipids would result in severe damage, especially during a process such as erythropoiesis, with each red cell processing the heme required for over 300 million hemoglobin molecules. Additionally, many of heme synthesis precursors and heme breakdown products are also toxic, and thus careful control of heme synthesis, trafficking, and degradation is a prerequisite.

Given these chemical and biochemical properties of heme, it is apparent that a system of transporters and chaperones would be necessary to efficiently and specifically distribute heme to hemoproteins found in various compartments in the cell, all the while preventing adventitious heme toxicity.

## REQUIREMENTS AND MECHANISMS FOR HEME DELIVERY TO SUBCELLULAR COMPARTMENTS

**FIGURE 1:**
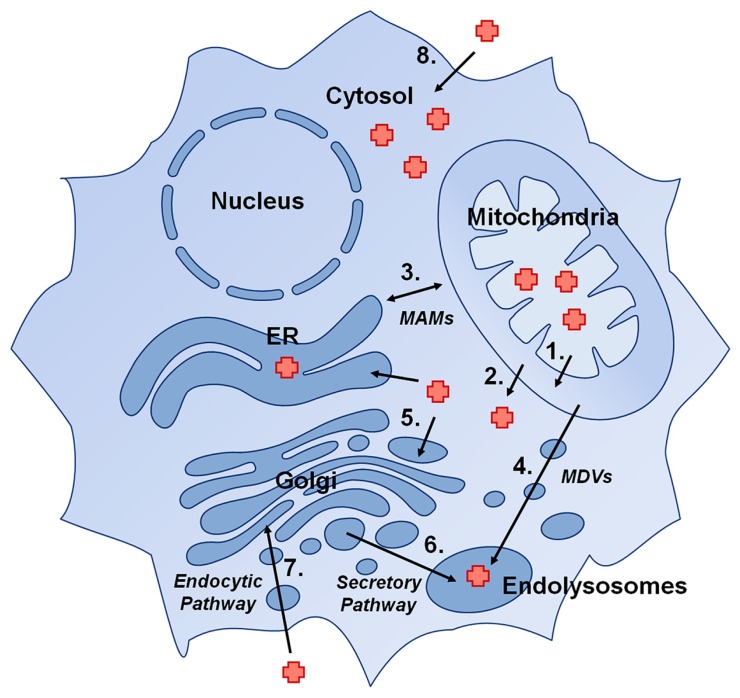
**Presumptive pathways for delivery of heme to hemoproteins in eukaryotic cells:** (1) Heme transport across the inner mitochondrial membrane, (2) Heme transport across the outer mitochondrial membrane, (3) direct heme trafficking from the mitochondria to the ER via mitochondria-associated membranes (MAMs), (4) trafficking of heme from mitochondria to endolysosomal compartments via mitochondrial-derived vesicles (MDVs), (5) transport of cytosolic heme into the secretory pathway by a heme exporter, (6) trafficking of heme in ER/Golgi to endolysosomal compartments, (7) import of heme into the cell via an endocytic pathway, and (8) import of heme via a plasma membrane heme importer.

Hemoproteins can be found in virtually every subcellular compartment of eukaryotic cells. In this section we will outline the requirement for heme delivery from its site of synthesis in the matrix, to the cytosol, to the secretory compartment, as well as other possible direct shuttling routes between organelles (**Figure 1**).

### HEME REQUIREMENTS IN THE MITOCHONDRIA

Most of the mitochondrial respiratory chain complexes rely on the redox capability of a heme prosthetic group (**Table 1**). Heme is present in these complexes in the *a*, *b*, or *c* forms. Heme *b* is the most common form of heme. It is the form synthesized by ferrochelatase, containing a C2 vinyl group and C8 methyl group, and is attached to proteins via non-covalent coordination of the heme iron with amino acid side chains (Gonzales and Neupert, 1990; Hederstedt, 2012). Heme *a* is modified from heme *b* and its porphyrins ring contains a C2 hydroxyethylfarnesyl and a C8 formyl side group (Hederstedt, 2012). Heme *c* is found covalently linked to proteins; the two vinyl side chains are converted to thioether linkages to cysteine residues in the apoprotein (Bowman and Bren, 2008).

**Table 1:** List of selected eukaryotic hemoproteins.

Hemoprotein	Function	Subcellular localization
Succinate dehydrogenase (Complex II)	Electron transport	Mitochondrial (IMM)
Cytochrome *bc*_1_ complex (Complex III)	Electron transport	Mitochondrial (IMM)
Cytochrome *c*	Electron transport	Mitochondrial (IMS)
Cytochrome *c* oxidase (Complex IV)	Electron transport	Mitochondrial (IMM)
CYP11A1	Steroid synthesis	Mitochondrial (matrix)
Hemoglobin	Gas-binding	Cytosolic
Myoglobin	Gas-binding	Cytosolic
Neuroglobin	Gas-binding	Cytosolic
Cytoglobin	Gas-binding	Cytosolic
Nitric oxide (NO) synthase	NO signaling	Cytosolic
Soluble guanylyl cyclases	NO and Ca^2+^ signaling	Cytosolic
Hap1p	Transcription factor	Nuclear
Bach1	Transcription factor	Cytosolic/nuclear
Rev-erb-α	Transcription factor	Nuclear
DGBR8	miRNA processing	Nuclear
mPer2	Circadian rhythm	Cytosolic/nuclear
Cytochrome *b*_5_	Electron carrier	Cytosolic
P450 cytochromes	Oxidation of metabolites and xenobiotics	ER membrane (active site is cytosolic)
Prostaglandin synthases (COX1 and COX2)	Autocrine/paracrine signaling	ER
Myeloperoxidase	Microbicide	Lysosome-like azurophil granules
Catalase	Antioxidant	Peroxisomes
Thyroperoxidase	Thyroid hormone synthesis	Plasma membrane
Ferric reductase	Iron transport	Plasma membrane
Lactoperoxidase	Microbicide	Secreted
Ligninase	Nutrient breakdown	Secreted
Heme-containing chloroperoxidase	Haloperoxidase	Secreted
Peroxidasin	Extracellular matrix synthesis	Secreted
HRG-3	Heme mobilization	Secreted
*Rhodnius* heme-binding protein (RHBP)	Heme mobilization	Secreted

The biogenesis and cofactor requirements of the mitochondrial respiratory complexes have recently been reviewed, but will be briefly discussed here with regard to heme transport and trafficking (Kim et al., 2012; Smith et al., 2012; Soto et al., 2012). The cytochrome *bc*_1_ complex (also known as coenzyme Q-cytochrome *c* reductase or Complex III) is a multi-subunit protein located with the other electron transport complexes in the inner mitochondrial membrane (IMM). The cytochrome *bc*_1_ complex includes two heme *b*-containing cytochromes as well as a heme *c*-containing cytochrome, and interacts with another hemoprotein, the electron carrier cytochrome *c*, for the purpose of generating a proton gradient along the IMM. Cytochrome *c* is soluble in the intermembrane space (IMS) of mitochondria and is found loosely associated with the IMM, as it carries an electron from the cytochrome *bc*_1_ complex to cytochrome *c* oxidase (COX). COX (also known as Complex IV) includes two heme *a*-containing cytochromes, which also participate in electron transfer and proton translocation into the IMS. Succinate dehyrogenase (Complex II) also contains a heme *b* moiety, which is actually required for stability of the complex (Lemarie and Grimm, 2009). The heme *b* in succinate dehydrogenase may also be involved in electron transport, but the exact function of this heme remains unclear (Kim et al., 2012).

In eukaryotes, the cytochrome *bc*_1_ complex (Complex III) is composed of seven or eight nuclear-encoded subunits that function together with three catalytic core proteins encoded in the mitochondrial genome (Xia et al., 1997; Iwata et al., 1998; Lange and Hunte, 2002). Cytochrome *b* is a membrane-bound core protein that contains two heme groups, each of which has a unique redox potential (Zhang et al., 1998). Heme *b*_H_, the high potential heme, is bound in an accessible cavity on the matrix side of the protein. Heme *b*_L_, the low potential heme, is found within the IMS portion of cytochrome *b*. Heme *b*_H_ can be inserted into cytochrome *b* without having to traverse a membrane; it is unknown whether or not a chaperone assists in this process (Brasseur et al., 1996). It is unclear if heme *b*_L_ is acquired in the matrix, or if this heme is inserted in the IMS, thus necessitating movement of heme across the IMM (**Figure 1**, Pathway 1). Cytochrome *c*_1_, being nuclear-encoded, is targeted to the mitochondria after being translated in the cytosol. Its heme cofactor is covalently attached in the IMS by the heme lyases Cyc3 and Cyt2 in yeast or holocytochrome c synthase (HCCS) in mammals (Bernard et al., 2003). In addition to the heme in cytochrome *c*_1_ and possibly the heme *b*_H_ of cytochrome *b*, the soluble respiratory protein cytochrome *c* also acquires its heme in the IMS from HCCS (Bernard et al., 2003).

The biogenesis of COX (Complex IV) in highly conserved in eukaryotes (Soto et al., 2012). Heme *a* in COX is bound to the mitochondrially encoded COX1 subunit. Heme *a* is synthesized via the conversion of heme *b* to the intermediate heme *o* by the farnesylation of the C2 position by COX10 (heme *o* synthase), and the subsequent oxidation of the C2 methyl side chain to formyl by COX15 (heme *a* synthase; Hederstedt, 2012; Soto et al., 2012). Both COX10 and COX15 are integral membrane proteins located in the IMM. Studies have indicated that insertion of heme *a* into COX1 occurs post-translationally, as early intermediates of the COX assembly do not contain heme *a* and are able to form in the absence of heme *a* biosynthesis (Khalimonchuk et al., 2010). This is in contrast with later intermediates, which do not form without the presence of heme *a*. It remains unclear if heme chaperones exist to shuttle heme *b* from its site of synthesis to COX10, or to deliver heme *a* to the COX1 subunit. By contrast, multiple mitochondrial chaperones, including COX17, SCO1, and COX11, have been shown to act as chaperones for delivery of copper to the COX complex [Also reviewed in Soto et al. (2012)].

In addition to the respiratory chain complex proteins, another cytochrome, cytochrome P450 side chain cleavage enzyme (also known as CYP11A1), is also found in the mitochondrial matrix. This enzyme is expressed in steroid-synthesizing tissues such as the brain and adrenal glands and functions to convert cholesterol to pregnenolone, a precursor for mineralocorticoids, glucocorticoids, androgens, and estrogens (Simpson and Boyd, 1967). CYP11A1 catalyzes this first step in steroid-synthesis by initiating three sequential monooxygenase reactions of the cholesterol side chain: hydroxylation of C20 and C22 and then cleavage the C20–C22 bond to generate the steroid precursor pregnenolone and isocaproic aldehyde. While the crystal structures of both bovine and human CYP11A1 have recently been solved, nothing is known regarding how the heme cofactor is inserted into this important hemoprotein (Mast et al., 2011; Strushkevich et al., 2011).

### HEME REQUIREMENTS IN THE CYTOSOL

While heme moves from the mitochondrial matrix into the IMS for insertion into particular cytochromes, heme must also exit the mitochondria for insertion into hemoproteins which acquire heme in the cytosol (**Table 1**). The most well known cytosolic hemoproteins are the gas-binding globins, including hemoglobin, myoglobin, neuroglobin, and cytoglobin. Adult hemoglobin consists of two α and two β chains, each bound to a molecule of heme. Hemoglobin can be generated *in vitro* (for example, by heterologous expression in *Escherichia coli,* or by recombining purified apo-hemoglobin with heme) indicating that no specific chaperone is required for heme insertion. Because of its asymmetric side chains, each heme can be incorporated into hemoglobin in two orientations. The term “heme disorder” refers to mixtures of these two orientations within a subset of hemoglobin molecules (Brown, 1976). Interestingly, the amount of heme disorder differs between hemoglobin synthesized *in vivo* or *in vitro* (Santucci et al., 1990). This is a possible indication that stereoselective insertion of heme into hemoglobin occurs, although the mechanism for this phenomenon remains unclear.

Other categories of hemoproteins that acquire their heme in cytosol include nitric oxide (NO) synthases and the soluble guanylyl cyclases (a NO receptor), the nuclear HBPs Hap1p, DGCR8, mPer2, Bach1 and Rev-erb-α, as well as the soluble form of cytochrome *b*_5_. Even P450 cytochromes, the most well known hemoproteins in the ER, are anchored in the ER membrane with their heme-containing globular domain facing the cytosol (Black, 1992; Avadhani et al., 2011). Thus, cytochrome P450 proteins likely acquire their heme from the cytosolic face of the membrane.

### HEME DELIVERY TO CYTOSOLIC PROTEINS

Cytosolic hemoproteins can acquire their heme from two possible sources: heme synthesized *de novo* in the mitochondria, or heme imported from outside the cell. Heme synthesized in mitochrondria exits the active site pocket of ferrochelatase, which faces the matrix side of the IMM (Wu et al., 2001). Exactly how heme exits ferrochelatase remains unknown. It is possible that this heme immediately intercalates into the IMM or is actively transported across the IMM and then is removed into the IMS by a heme-binding chaperone and then moved across the OMM by a high affinity heme transporter (**Figure 1**, Pathways 1 and 2).

The sole mitochondrial heme exporter identified to date is the mitochondrial isoform of Flvcr1 (Quigley et al., 2004; Keel et al., 2008). Flvcr1, a member of the major facilitator superfamily of transporters, was initially identified as a plasma membrane heme exporter. Cats infected with feline leukemia virus, subgroup C (FeLV-C) develop aplastic anemia due to failure of erythroid differentiation of burst-forming units-erythroid (BFU-E) to colony-forming units-erythroid (CFU-E). Viral infection of these erythroid progenitors interferes with and possibly downregulates cell surface expression of the viral receptor, Flvcr1 (Quigley et al., 2004). Early experiments showed that Flvcr1 could mediate cellular efflux of either exogenously added or endogenous heme (Quigley et al., 2004; Yang et al., 2010). Mice lacking Flvcr1 die either at E7.5 or between E14.5 and E16.5. Similar to Hmox1, Flvcr1 is expressed in the yolk sac, ectoplacental cone, and placenta at E7.5. Thus the early death of Flvcr1 null embryos is likely due to heme accumulation within cells, although it is also possible that Flvcr1 is required for delivery of maternal heme. Keel et al. (2008) conclude that Flvcr1 null embryos die at the later stage due to impaired erythropoiesis. Red cells in these mice are blocked at the proerythroblast stage, in keeping with feline infection model as well as *in vitro* studies showing that K562 cells fail to differentiate when infected with FeLV-C (Keel et al., 2008). Interestingly, Flvcr1 mice exhibit cranial and limb deformities which are not usually associated with lack of definitive erythropoiesis, indicating additional functions for Flvcr1 in development.

Recently, Chiabrando et al. (2012) identified a mitochondrial isoform of Flvcr1, termed Flvcr1b. While Flvcr1a encodes a plasma membrane-localized transporter, Flvcr1b contains an alternative transcription start site, resulting in a shortened amino terminus containing a mitochondrial targeting signal. Depletion of Flvcr1b in HeLa cells results in accumulation of mitochondrial heme, indicating that Flvc1b plays a role in heme export from the mitochondria (Chiabrando et al., 2012). Specific deletion of Flvcr1a in mice resulted in death between E14.5 and birth due to hemorrhage, edema, and skeletal abnormalities; however, erythropoiesis in these mice appeared to be normal and fetal liver cells from these mutant mice were able to repopulate irradiated wild type mice (Chiabrando et al., 2012). The authors conclude that Flvcr1a null mice suffer mainly from the loss of a plasma membrane heme exporter. Heme overload in endothelial cells leads to a loss of vascular integrity resulting in the hemorrhage, edema, and skeletal defects observed in these mice. The authors attribute the lethality of the original Flvcr1 knockout mice to lack of heme export from the mitochondria by Flvcr1b, as mice lacking Flvcr1a but not mitochondrial Flvcr1b appear to have normal erythropoiesis.

Numerous questions regarding heme exit from the mitochondria remain unanswered. It is not yet known if Flvcr1b resides on the mitochondrial IMM or OMM, with the possibility of another distinct mitochondrial heme exporter yet to be found. It is also unclear whether Flvcr1b is actually located within the mitochondria or is associated with membranes that tether the mitochondria to other organelles. If mitochondrial heme export is indeed attenuated, how do *Flvcr1*^-^^/^^-^ embryos even survive until E14.5? Does Flvcr1b function in specific cell types? Yeast do not appear to have an obvious FLVCR homolog, yet are able to export heme from the mitochondria; what alternate mechanisms exist for mitochondrial heme export? No specific heme chaperones have been found to bind heme in the IMS, or to deliver heme to cytosolic hemoproteins. A number of cytosolic HBPs have been identified, including 22- and 23-kDa HBPs and the HBP homolog, SOUL (Iwahara et al., 1995; Taketani et al., 1998; Zylka and Reppert, 1999). These proteins could serve as “sponges” for heme exiting the mitochondria, simultaneously protecting the cell from heme’s cytotoxicity and also delivering heme to target hemoproteins. It remains unclear whether multiple non-specific porphyrin-binding molecules perform the role of chaperoning heme in the cytosol, or if there are heme- and/or hemoprotein-specific pathways.

Presumably, heme imported into the cell, rather than solely heme exported from the mitochondria, could be bound by cytosolic heme chaperones and delivered to newly forming hemoproteins (**Figure 1**, Pathway 8). As such, plasma membrane heme importers could also be sources of heme for these enzymes. A discussion of putative heme importers can be found in the accompanying review of heme in pathophysiology by Chiabrando et al. (2014). Here we will focus on the heme importer, Hrg1.

The heme-responsive paralogs, *hrg-1* and *hrg-4*, were initially discovered as transmembrane domain-containing permeases upregulated in response to heme deficiency in *Caenorhabditis elegans* (Rajagopal et al., 2008). HRG-4 is localized to the apical plasma membrane of the worm intestine and serves to import dietary heme into the animal (Rajagopal et al., 2008; Yuan et al., 2012). HRG-1 localizes to lysosome-like vesicles in the intestine, and serves to mobilize heme stored in these compartments (Rajagopal et al., 2008; Yuan et al., 2012). While *hrg-4* does not appear to have homologs in other species, *hrg-1* homologs, also called solute carrier 48 A1 (SLC48A1) proteins, have been found in diverse organisms, including *Leishmania* spp., zebrafish, and mammals (Rajagopal et al., 2008; Huynh et al., 2012; Yuan et al., 2012; White et al., 2013). Knockdown of *hrg-1* in zebrafish leads to anemia, although the genetic etiology for the anemia phenotype is not known. Transport of heme by *hrg-1* and *hrg-4* has been demonstrated directly using electrophysiological currents in *Xenopus* oocytes, and indirectly using heme-dependent growth of *C. elegans* and *Saccharomyces cerevisiae* or uptake of heme analogs as indicators of heme import.

Mammalian Hrg1 has been shown to be expressed widely, with the highest expression in the brain, heart, kidney, and muscle, with some expression in the placenta and intestine (Rajagopal et al., 2008). Hrg1 has been linked to a possible role in cancer progression, as its interaction with V-type ATPases is associated with changes in endocytic trafficking, extracellular acidification, altered glucose metabolism, and matrix metalloprotease activity (O’Callaghan et al., 2010; Fogarty et al., 2013). Depletion of Hrg1 in MCF7 cells resulted in decreased invasiveness and migration capabilities in these cells. Hrg1 has also been found to be a target of nuclear factor (erythroid-derived 2)-like 2 (Nrf2), the antioxidant response transcription factor (Campbell et al., 2013). In independent studies, human Hrg1 colocalized with Lamp1 in lysosomes, and Hrg1 is found on the erythrophagolysosome in macrophages during RBC heme-recycling (Delaby et al., 2012; White et al., 2013). Hrg1 has been shown to mediate hemoglobin-derived heme export from this compartment in macrophages and this process will be discussed in Section “Heme Transport during Erythropoiesis.”

### HEME REQUIREMENT IN THE SECRETORY PATHWAY

A variety of hemoproteins function in multiple components of the secretory pathway (**Table 1**). Lysosomal, peroxisomal, plasma membrane-targeted and secreted hemoproteins are folded and processed within the ER and Golgi, and thus the cell must have a means of transferring heme from the mitochondria into various subcellular membrane-bound compartments. These hemoproteins include the well known prostaglandin synthases (COX1 and COX2) in the ER, myeloperoxidase (MPO) in lysosome-like azurophil granules, eosinophil peroxidase in eosinophil granules, catalase in peroxisomes, the plasma membrane proteins thyroperoxidase and ferric reductase, as well as numerous secreted proteins including lactoperoxidase, fungal ligninase and chloroperoxidase, *C. elegans hrg-3*, *Drosophila* peroxidasin, and RHBP (*Rhodnius* heme-binding protein) in the blood-feeding insect *Rhodnius prolixus*.

Almost nothing is known regarding how these proteins obtain their heme cofactors. Given that the secretory and endocytic pathways are somewhat contiguous, with protein and lipid components being shuttled from location to another, it is feasible that heme need only be transported across a single membrane into the lumen of one such compartment, and from there can be mobilized to its intended destination by carrier proteins (**Figure 1**, Pathways 3–7; De Matteis and Luini, 2008). For example, heme exiting the mitochondria may be transported into the ER, and from there be inserted into its target hemoprotein during protein folding, following which the protein can then traffic to its target organelle. It is also possible that one or many heme chaperones exist in the secretory pathway for delivery of heme to hemoproteins in their destination compartments. The fact that no mechanism for targeting heme to the secretory pathway has been discovered suggests that multiple mechanisms exist and can compensate for loss of a single such constituent.

In contrast to many hemoproteins in the secretory pathway, the maturation process of MPO has been well-characterized. MPO is a microbicidal protein generated by myeloid cells; its ability to chlorinate substrates enables it to generate hypochlorous acid from hydrogen peroxide [Reviewed in Klebanoff et al. (2013)]. This hemoprotein is extensively processed as it moves through the ER and Golgi; a single 80 kDa apoproMPO peptide is converted to a glycosolated, heme-containing protein consisting of a 59 kD heavy subunit and a 15.5 kD light subunit that is targeted for secretion in its pro-form or for storage in azurophil granules as a mature protein [Reviewed in Hansson et al. (2006)]. Early studies showed that disruption of the Golgi stacks with brefeldin A treatment resulted in MPO that is able to acquire heme but was improperly processed and remained in the pro-form (Nauseef et al., 1992). Moreover, treatment of the cells with succinyl acetone to inhibit heme synthesis also resulted in MPO that was improperly processed, while the processing and trafficking of other lysosomal proteins remained unchanged. The authors concluded that cleavage of MPO into its mature heavy and light subunits occurred in a post-ER compartment, that insertion of heme occurred in the ER, and that heme insertion was required for further processing. Further support for these conclusions was provided by mutants, such as the R569W MPO mutant which is unable to acquire heme and remains trapped in an apo-form in the ER (a result suggesting that heme insertion is necessary for ER exit), and the Y173C MPO mutant, which does acquire heme but is unable to exit the ER (suggesting that heme insertion is mediated within the ER; Nauseef et al., 1994; DeLeo et al., 1998). The aberrant processing of these mutant forms of MPO is especially informative, as these results lack the confounding effects of brefeldin A treatment.

The ER chaperones calreticulin and calnexin, which perform quality control during the folding of glycoproteins, unsurprisingly bind MPO as it is being folded and processed in the ER. Interestingly, these two similar proteins are able to distinguish between processed and unprocessed MPO, in a manner that likely enables sequential interaction of MPO with both chaperones. Calreticulin appears to bind apoproMPO preferentially, while calnexin binds both apoproMPO and the heme-containing apoMPO (Nauseef et al., 1998). It is possible that calnexin plays a role in the insertion of heme into MPO as it binds to both the apopro- and heme-containing pro- versions of the protein. Interestingly, one study showed that overexpression of calnexin in the fungus *Aspergillus niger* resulted in increased production of a secreted hemoprotein, manganese peroxidase, but only in the absence of heme supplementation in the media. This suggested that calnexin can function as a protein chaperone capable of enhancing holo manganese peroxidase assembly (Conesa et al., 2002). Studies have shown that palmitoylation of calnexin increased its localization to mitochondrial-associated membranes (MAMs) of the ER (Lynes et al., 2012) – a location where heme could, in principle, enter the ER (as discussed in Section “Mechanisms for Heme Delivery to the Secretory Pathway”). It has very recently been shown that palmitoylation switches calnexin from an ERp47-interacting quality control molecule to a MAM-associated regulator of ER-mitochondrial crosstalk and two proteomic analyses of MAMs have independently detected the presence of calnexin (Lynes et al., 2013). While it is tempting to speculate that calnexin could moonlight as a chaperone for heme entering the ER from the mitochondria, the matter remains a mystery until further studies are performed.

### MECHANISMS FOR HEME DELIVERY TO THE SECRETORY PATHWAY

Theoretically, there are a number of ways heme could enter the lumen of the secretory pathway. Heme released from the mitochondria could pass through the cytosol to be actively transported across the membrane of any subcellular organelle, (although no such transporter has yet been identified; **Figure 1**, Pathway 5). Heme imported into the cytosol could be moved into the secretory pathway in this manner as well. Heme entering the cell through an endocytic process (i.e., heme bound to hemopexin internalized via the low-density lipoprotein receptor-related protein LRP/CD91) could be trafficking to such compartments, with the possible participation of a chaperone to sequester and guide heme to its destination (**Figure 1**, Pathway 7). One appealing possibility is that nascent heme synthesized in the mitochondria could be shielded by membranes using vesicular trafficking or by entering the secretory pathway through direct contacts between the ER and mitochondrial membranes (**Figure 1**, Pathways 3 and 4).

Contacts between ER and mitochondrial outer membranes were observed over 40 years ago (Ruby et al., 1969; Lewis and Tata, 1973). Jean Vance coined the term MAM when she identified a functional relationship between these organelles to exchange phospholipids (Vance, 1990). With better subcellular fractionation techniques, and the advent of fluorescent imaging, especially the recent development of superresolution imaging, dynamic microdomains where mitochondrial membranes are tethered to ER membranes have been observed. These include the ER-mitochondrion encounter structure (ERMES) in yeast and the previously mentioned MAMs, found in plants and animals (Kornmann et al., 2009, 2011). These structures are involved in facilitating the transport of ionic calcium into the mitochondria, regulation of autophagy and apoptosis, and, more relevant to this review, the trafficking of lipids (Michel and Kornmann, 2012). The transport of phosphatidylserine from the ER to mitochondria has been demonstrated and it is hypothesized that other lipids are mobilized between these two organelles in a similar manner. Interestingly, two independent proteomic analyses of MAMs have detected the presence of coproporphyrinogen III oxidase, ferrochelatase, HBP 1, and heme oxygenase 2 (Poston et al., 2011; Zhang et al., 2011). It is intriguing to postulate that these ER-mitochondria connections could be an axis for heme transport (**Figure 1**, Pathway 3).

Another possible mechanism for the trafficking of heme from the mitochondria to other organelles is the use of mitochondrial-derived vesicles (MDVs), which have been shown to traffic to both peroxisomes and lysosomes (**Figure 1**, Pathway 4; Neuspiel et al., 2008; Schumann and Subramani, 2008). Initially, 70–100 nm vesicles were shown to deliver specific mitochondrial cargo proteins to peroxisomes. Other vesicles were later observed carrying cargo to lysosomes in response to increase in cellular oxidative stress (Soubannier et al., 2012; McLelland et al., 2014). As the processes of heme synthesis, mitochondrial respiration, and responding to oxidative stress are innately coupled by their shared metabolic pathways and mitochondrial location in the cell, it is possible to speculate that heme may be mobilized to these organelles via this mechanism.

## HEME TRAFFICKING AND IRON METABOLISM

### HEME TRANSPORT DURING ERYTHROPOIESIS

**FIGURE 2:**
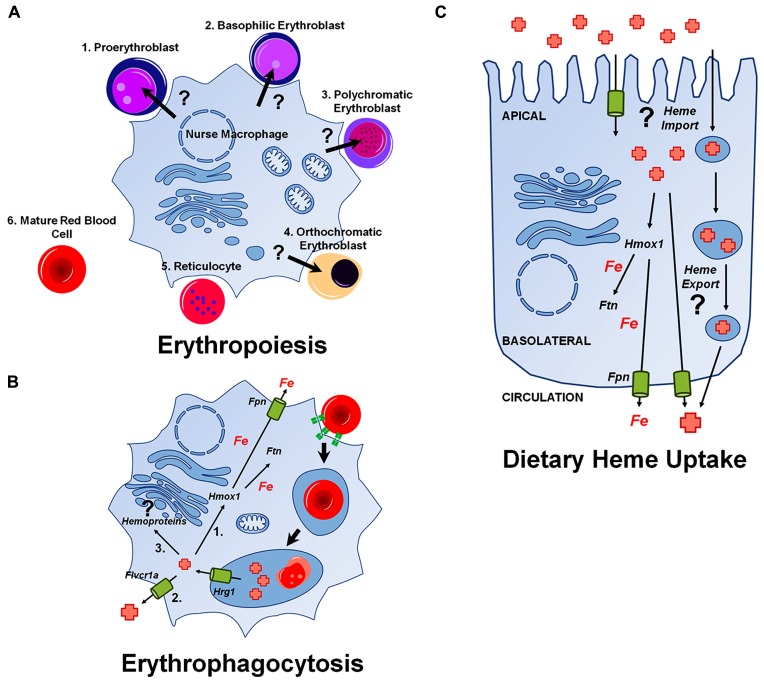
**Heme transport pathways relevant during major physiological events.** Unknown pathways are indicated with a (?). **(A)** Putative delivery of heme from a central nurse macrophage to developing erythroblasts. **(B)** Pathways for iron and heme-recycling during erythrophagocytosis. Hrg1 imports heme into the cytoplasm from the erythrophagolysosome. A portion of heme is (Green et al., 1954) degraded by Hmox1 and iron released is either stored in ferritin (Ftn) or exported by ferroportin (Fpn; Kendrew et al., 1958). Some heme may be exported via a plasma membrane heme transporter such as Flvcr1a, or (Cannon et al., 1984) used by the cell for incorporation into hemoproteins. **(C)** Pathways for dietary heme uptake by enterocytes. There is evidence for an intestinal heme import across the apical membrane of enterocytes, although it is unclear whether this pathway utilizes membrane-bound transporters or endocytic uptake or both mechanisms. Most heme is mobilized into the circulation as iron after degradation by Hmox1, but heme may, in principle, also directly enter the circulation.

During definitive erythropoiesis, hematopoietic progenitors differentiate into mature RBCs. In the fetal liver or bone marrow, hematopoietic stem cells develop into early BFU-E progenitors and then into CFU-E progenitors, which are both defined by their capacity to form cell clusters *in vitro* [Reviewed in Palis (2014)]. These progenitor cells progress into erythroid precursors, which develop from early proerythroblasts (ProE), to basophilic erythroblasts (Baso), to polychromatophilic erythroblasts (PolyE), and finally become orthochromatic erythroblasts (OrthoE). Orthochromatic erythroblasts enucleate to form reticulocytes which move out into circulation and after about 24 h become mature RBCs. The erythroid precursors develop from the ProE stage to the OrthoE stage in a unique niche called the erythroblastic island. This niche consists of a central nurse macrophage surrounded by a ring of developing RBC precursors. It is at this stage where heme and hemoglobin synthesis rapidly increase, as the cells draw near their fate as RBCs (**Figure 2A**).

Nurse macrophages maintain physical contacts with erythroblasts as they differentiate. They phagocytose the nuclei extruded by red cells, and are thought to enhance differentiation in some capacity. This specialized niche has been shown to be dispensable for erythropoiesis under steady state conditions (Chow et al., 2013; Ramos et al., 2013). However, in response to anemia, erythropoiesis expands fromthe bone marrowto the spleen and liver,with increased iron mobilization and RBC formation. Recent studies have shown that nurse macrophages play a regulatory role during stress erythropoiesis. Depletion of macrophages does not alter the numbers or profiles of erythroid cells in normal mice. However, mice with phlebotomy-induced anemia showed a decreased ability to recover from this state in the absence of macrophages, as shown by decreased red cell formation compared to control mice. Interestingly, the loss of these macrophages ameliorated symptoms of both polycythemia vera and β-thalassemia, as both diseases involve a pathological increase in RBC production (Hashimoto et al., 2013; Ramos et al., 2013).

Some have suggested that nurse cells may contribute nutrients, possibly even iron or heme itself, to developing erythroblasts [Reviewed in Manwani and Bieker (2008)]. Under steady state conditions, the contribution of nurse macrophages is clearly dispensable for normal erythropoiesis; however, under stress erythropoiesis, the nutritional contribution of nurse macrophages may play a more important role. Developing erythroblasts co-cultured with macrophages in transwells showed a marked decrease in the beneficial effects that physical contact between the two cell types normally provides (Ramos et al., 2013). This result indicates that direct contact between the two cell types is required to maximize the positive effect of macrophages, but does not rule out a role for secreted factors.

It was reported that ferritin molecules were localized between the membranes of the central nurse macrophage and developing erythroblasts, and that this ferritin is taken up the erythroblasts by micropinocytosis (Policard and Bessis, 1962). However, the original source of this ferritin remains a mystery. It is possible that a portion of heme and/or iron within a maturing red cell is derived from inter-cellular transport from macrophages to support the initial stages of hemoglobinization, especially under stress conditions. In support of this hypothesis, heme importers Hrg1 and exporters Flvcr1 are both expressed in red cells and macrophages.

### HEME MOBILIZATION DURING ERYTHROPHAGOCYTOSIS

Much like the birth of RBCs, the final moments of a red cell are spent in the care of a macrophage. Human RBCs have a lifespan of about 120 days, after which they become senescent and are recycled by macrophages of the reticuloendothelial system (RES) found in the spleen and liver [Reviewed in Knutson and Wessling-Resnick (2003)]. This process – phagocytosis of RBCs and the breakdown of billions of molecules of hemoglobin – is termed erythrophagocytosis (EP; **Figure 2B**). EP is essential, as the bulk of iron required for the synthesis of new hemoglobin (~25 mg/day) derives from RBC recycling (Bratosin et al., 1998). By contrast, only about 1 mg per day of dietary iron contributes to erythropoiesis. Heme oxygenase 1 (Hmox1) plays a vital role in this process, freeing iron from its protoporphyrin ring, and enabling the release of this iron for reuse. *Hmox1*^-^^/^^-^ mice suffer from anemia, reduced serum iron, and accumulation of iron in the spleen and liver, as the ability to recycle heme-iron in these mutants is almost completely crippled (Poss and Tonegawa, 1997). *In vitro* experiments showed that *Hmox1*^-^^/^^-^ macrophages die when fed RBCs, and that splenic and liver macrophages are absent in *Hmox1*^-^^/^^-^ mice (Kovtunovych et al., 2010).

Heme extracted from RBCs was postulated by some to be degraded by Hmox1 in the phagolysosome (Beaumont and Canonne-Hergaux, 2005). Subsequently, the newly released iron was either stored in ferritin (Ftn) or returned to the circulating pool of iron via the cellular heme exporter, ferroportin (Fpn). A major problem with this model is that Hmox1, while localizing to the ER via a C-terminal transmembrane segment, has been shown to be oriented with its active site facing the cytosol (Gottlieb et al., 2012). Furthermore, the pH optimum for heme catabolism by Hmox1 is closer to physiological pH (pH 7.6) than lysosomal pH, and maximal Hmox1 activity requires the presence of biliverdin reductase (Liu and Ortiz de Montellano, 2000; Reed et al., 2010). Studies have shown that heme oxygenases are not present on the phagolysosomal membrane, even though this membrane is partially derived from the ER (Delaby et al., 2012). Thus Hmox1 likely does not have access to heme within the phagolysosomal compartment, suggesting that heme must exit this membrane compartment for degradation.

Hrg1, which has been shown to localize to endocytic compartments in mammalian cells, has recently been reported to transport heme across the phagolysosomal membrane (Delaby et al., 2012; White et al., 2013). Hrg1 is expressed in RES macrophages and is upregulated at both the mRNA and protein level in the presence of heme and during EP. This was true in both an *ex vivo* model using bone-marrow derived macrophages (BMDMs) treated with damaged RBCs, as well as in the livers of mice treated with either heme, damaged RBCs, or the hemolysis agent phenylhydrazine (White et al., 2013). During EP, Hrg1 accumulates on the membrane of the phagolysosome (White et al., 2013), and when Hrg1 is depleted by siRNA, BMDMs are incapable of upregulating the machinery normally required to deal with the influx of heme and iron (including Hmox1, Ftn, Fpn, and Hrg1 itself) indicating a lack of heme export from the phagolysosome.

Interestingly, a polymorphism of Hrg1 (P36L mutation) associated with anemia in four patients was shown to be defective in heme export in both a yeast model of heme transport, and during EP in BMDMs (White et al., 2013). These experiments used a heterogously expressed hemoprotein reporter, horseradish peroxidase (HRP), targeted to the Golgi to interrogate cellular heme availability and showed that heme derived from recycled RBCs could be incorporated into an apohemoprotein by assaying HRP activity in BMDMs during EP. While further experiments are required to definitively show that the increased HRP activity was due to incorporation of heme recycled *in toto,* rather than endogenously synthesized heme, these experiments suggest that not all heme liberated during EP is degraded via Hmox1, and that a portion can be reused by the cell.

### DIETARY HEME TRANSPORT AND ENTEROCYTE METABOLISM

Heme is the most bioavailable form of iron in the diet, as ferric iron can form insoluble ferric hydroxide or hydroxyl-iron dimers in the intestine and the uptake of soluble iron is inhibited by iron chelators found in certain foods [Reviewed in West and Oates (2008)]. Early studies using radiolabeled heme showed that heme enters enterocytes, where most of it is degraded and exported as iron to the circulation (**Figure 2C**; Walsh et al., 1955; Weintraub et al., 1965; Brown et al., 1968). Interestingly, these early studies showed that unlike uptake of inorganic iron, heme uptake is not significantly upregulated during iron deficiency (Turnbull et al., 1962). Later studies showed that this process was mediated by a receptor or transporter, as it was pH dependent, saturable, and that its activity was lost in the presence of trypsin (Grasbeck et al., 1979, 1982; Galbraith et al., 1985; Majuri, 1989; Noyer et al., 1998). Studies using zinc mesoporphyrin showed that uptake was inducible with heme depletion and specific to certain cell types, as it was observed in intestinal Caco-2 and I-407 cells and hepatic HepG2 cells but not in mouse fibroblasts (Worthington et al., 2001).

A recent study analyzed the uptake of ^58^Fe-heme versus a non-heme iron source (^57^FeSO_4_) in pregnant women (Young et al., 2012). Cord blood from neonates taken at time of delivery contained significantly more ^58^Fe than ^57^Fe. This study suggests that iron absorbed in the form of heme contributes substantially to body iron stores presumably because dietary heme-iron is more bioavailable for intestinal absorption and therefore is preferentially delivered to the developing fetus. However, it is unclear whether the ^58^Fe detected is due to degradation of dietary heme within the intestine or, more provocatively, that a portion of ingested ^58^Fe-heme is delivered intact to the fetus. To differentiate between these possibilities, similar studies must be undertaken using heme labeled in the porphyrin ring.

The relative contributions of receptor-mediated endocytosis versus membrane transport to intestinal heme uptake remain unclear. Early studies of heme uptake in dogs and rats used diaminobenzidine (DAB) staining to visualize intact heme taken up into intestinal epithelial cells (Parmley et al., 1981; Wyllie and Kaufman, 1982). Heme was shown to accumulate on the surfaces of microvilli and in endosomal compartments in the apical cytoplasm, evidence for uptake via an endocytic process. However, these studies do not exclude the possibility that heme is also directly transported across the apical plasma membrane, as heme accumulating in the cytosol may not be sufficiently concentrated to visualize with DAB staining.

It was reported that heme carrier protein 1 (HCP1) was an intestinal heme importer (Shayeghi et al., 2005). Later, biochemical and genetic studies determined that HCP1 was a high affinity folate transporter, as it was capable of mediating folate transport and loss-of-function mutations in human patients were associated with hereditary folate malabsorption (Qiu et al., 2006). Qiu et al. (2006) note that folate supplements completely corrected any hematological defects associated with folate deficiency, indicating that this syndrome was not caused by defective heme uptake. An analysis of transport by HCP1, now termed HCP1/proton coupled folate transporter (PCFT), supported the conclusion that the physiological substrate of HCP1/PCFT is folate, though the protein may have weak heme transport activity. Thus, the mechanism (or mechanisms) of intestinal heme import remains unknown.

It may be useful to apply our knowledge of better-characterized paradigms of intestinal import to study intestinal heme uptake. For example, does heme traverse enterocytes via transcytosis, in a manner similar to vitamin B_12_, which is endocytosed in clathrin-coated vesicles, trafficked to lysosomes, and then secreted from the basolateral intestinal membrane? Does heme, like iron, utilize a transcellular trafficking route, making use of apical importers and basolateral exporters for directed movement into the body? Additionally, early studies showed that the bulk of heme exported from the intestine into the portal circulation is in the form of free iron released from degraded heme. What if heme is not exported from the enterocytes via blood? It is worth noting that cholesterol, another bulky, hydrophobic molecule, is exported into the lymph after being absorbed in the intestine (Abumrad and Davidson, 2012).

### HEME TRAFFICKING DURING EMBRYOGENESIS

It has long been known that metal homeostasis plays a vital role in embryonic and post-embryonic development (Kambe et al., 2008). For example, mouse pups from iron- or copper-depleted dams experience developmental and neurological abnormalities whose severity is dependent on the timing and extent of the maternal deficiency. A number of metal transporters are embryonic lethal when targeted for gene knockout. There are two separate but interconnected processes that should be taken into account when discussing this topic: (a) metal transport within embryonic cells as pluripotent cells grow and differentiate into tissues; and (b) maternal transfer of metals to developing embryos that lack an independent dietary source for these nutrients. We will consider the roles of both modes of transport with regards to heme homeostasis.

The respective phenotypes of the Flvcr1 and Flvcr1a knockout mice provide some insight into the role of heme transport during embryogenesis. As previously mentioned, Keel et al. (2008) report that homozygous Flvcr1 knockout pups (which lack both the mitochondrial and plasma membrane isoforms of Flvcr1) are found dead at either E7.5 or later at E14.5–E16.5. The authors infer from this observation that Flvcr1 is required for definitive erythropoiesis (which begins at ~E12) and not embryonic erythropoiesis. In support of this, they mention that yolk sac-derived erythroblasts do not express Flvcr1 and appear normal in *Flvcr1*^-^^/^^-^ mice. If the cause of mortality in *Flvcr1*^-^^/^^-^ mice is defective heme export from the mitochondria, then we must wonder about the source of heme used for embryonic erythropoiesis. It may be that this heme is maternally derived via one or more heme importers in the early embryo. It is known that homozygous ferrochelatase knockout embryos can be detected at the E3.5 preimplantation stage, but are reabsorbed and undetectable at E9–E10 – a phenotype also observed for uroporphyrinogen decarboxylase knockout animals (Phillips, personal communication; Magness et al., 2002; Phillips et al., 2007). Presumably, the ferrochelatase null phenotype should overlap with the *Flvcr1*^-^^/^^-^ phenotype, as both would cause heme deficiency in the cell. The greater severity of the ferrochelatase mutant hints that, in the *Flvcr*^-^^/^^-^ mice, a small portion of heme can exit the mitochondria in an Flvcr1-independent manner to sustain life for a few extra days.

Interestingly, deletion of the Flvcr1a isoform also causes embryonic lethality (Chiabrando et al., 2012). This lethality, due to hemorrhages, edema, and skeletal malformations, is observed later during development, usually between E14.5 and birth. Chiabrando et al. (2012) speculate that many of these defects, also observed in mice with defects in endothelial integrity, are due to heme buildup within endothelial cells leading to widespread oxidative stress and hypoxic conditions in developing embryos. Interestingly, Flvcr1b is upregulated in these mice, presumably leading to increased cytoplasmic heme as evidenced by increased levels of Hmox1, though its activity is insufficient to rescue the embryos. Thus, plasma membrane heme export is an essential regulator of heme homeostasis during development.

While intercellular and intracellular heme transport are critical during embryogenesis, maternal transfer of heme to offspring may also play a critical role in embryonic development. This process has been demonstrated in *C. elegans*, where the small peptide HRG-3 serves as a chaperone for heme delivery to developing embryos and extraintestinal tissues (Chen et al., 2011). When the worm is heme deprived, expression of *hrg-3* in the intestine is upregulated >300-fold. HRG-3 is processed in the secretory pathway into a 45-amino acid chaperone which binds heme in a stoichiometry of 1:2 (heme:protein). Mature HRG-3 is secreted into the worm’s circulation and taken up by extracellular tissues and developing oocytes. When *hrg-3* null worms are grown under heme limiting conditions, they show embryonic lethality and delayed growth, indicating a role for *hrg-3* in the distribution of heme from the intestine during early embryonic and larval development (Chen et al., 2011).

It was not surprising to discover a mechanism for heme transport along a maternal-to-embryonic axis in *C. elegans*, as this animal is a heme auxotroph and thus completely reliant on dietary heme. Surprisingly, however, a similar pathway was found in the blood sucking insect *R. prolixus*, a heme prototroph (Walter-Nuno et al., 2013). In a single meal, *R. prolixus* can ingest several times its own weight in blood. To deal with the oxidative stress associated with such a massive amount of heme, *R. prolixus* expresses a 15 kDa protein termed RHBP (Oliveira et al., 1995). RHBP is synthesized in the insect’s fat body at all stages of development. It is secreted into the hemolymph, where it and any heme bound to it are taken up into developing oocytes via receptor-mediated endocytosis. Interestingly, silencing of RHBP results in a unique form of embryonic lethality. Eggs laid early during oviposition retain their characteristic red color and develop normally; however, eggs laid at the later time are not viable and are pale due to lack of heme (Walter-Nuno et al., 2013). These eggs showed evidence of fertilization, but no embryo formation. Additionally, analysis of both heme-dependent and heme-independent mitochondrial enzymes showed widespread mitochondrial dysfunction in these later eggs (Walter-Nuno et al., 2013).

*Rhodnius* eggs require maternal heme because they are incapable of producing sufficient endogenous heme to sustain development. The use of RHBP couples the process of ameliorating heme toxicity in the adult to embryonic development – turning a liability into an essential asset. Interestingly, the heme delivered to these eggs is not degraded to release iron (as no heme degradation products are detected in the eggs) and the heme is incorporated *in toto* into embryonic hemoproteins.

## EVIDENCE FOR HEME TRANSPORT BETWEEN CELLS AND TISSUES

While the requirement for intracellular heme trafficking, based on the understanding of heme chemistry and cell membrane dynamics, is fairly intuitive and supported by the well-established paradigm for copper, zinc and iron trafficking pathways, the concept of heme trafficking from one tissue to another is controversial. Why move heme from one tissue to another if each cell in an organism is capable of synthesizing its own heme? However, there are several lines to evidence to support the potential existence of these pathways:

(1) While the bulk of heme imported into the intestine is purported to be degraded by heme oxygenases and exported as iron, small amounts of heme have been show to be transported intact along the basolateral membrane of enterocytes. Studies in polarized monolayers of human enterocyte-like Caco-2 cells have shown that a portion of heme transported intact across the basolateral membrane (Uc et al., 2004). Basolateral transport of heme from intestinal cells was also observed in guinea pigs (Conrad et al., 1966).
(2) Patients suffering acute attacks of porphyrias are administered heme intravenously as therapeutic treatment. This treatment results in increased heme-dependent enzyme activities in the liver, indicating that exogenous heme can be utilized *in toto* (Puy et al., 2010).
(3) The existence of widely expressed mammalian plasma membrane heme importers and exporters implies a physiological role for heme import and export (Quigley et al., 2004; Keel et al., 2008; Rajagopal et al., 2008) For example, we have discussed the heme importer Hrg1, which localizes to the plasma membrane in polarized cells, and is expressed in a number of tissues including the brain, kidney, heart, skeletal muscle, liver, lung, placenta, and small intestine. Knockdown of Hrg1 in zebrafish causes severe anemia, indicating a possible role for intercellular heme transport during erythropoiesis.
(4) With specific regards to heme-recycling in macrophages of the RES, there is evidence that intact heme is exported from the cells after EP. After phagocytosis of ^59^Fe-labeled RBCs, ^59^Fe-labeled heme was released from J774 mouse macrophages (Knutson et al., 2005). Additionally, *Flcvr*-deleted macrophages showed impaired export of ^55^Fe-labeled heme (Keel et al., 2008).
(5) Lastly, while heme synthesis mutants are lethal, these embryos do survive for several days. For example, mice lacking ferrochelatase live beyond E3.5, and zebrafish lacking protoporphyrinogen oxidase survive for 35 days post-fertilization (Magness et al., 2002; Dooley et al., 2008). This implies that developing embryos can either develop in the absence of heme or plausibly mobilize heme stores during initial stages of development, independent of heme synthesis.

The severity of phenotypes associated with loss of heme synthesis indicates that intracellular heme trafficking pathways are not the primary modes to support the heme requirements of animals. The generation of (a) tissue-specific heme synthesis knockout animals; (b) genetic chimeras using *in vivo* tissue-specific reporters; and (c) new modalities of live imaging of heme using label-free microscopy at the tissue and subcellular level would be greatly beneficial in determining the capacity for inter- and intra-tissue heme transport. Ultimately, these pathways may be relevant under specific conditions of aberrant iron or RBC homeostasis, or during pathogenesis. We include in this review a call for further testing and analysis of these ideas.

## CONCLUSION

In this review, we have discussed the following topics:

• The biophysical and physiological need for heme transport across biological membranes
• A detailed catalog of hemoproteins found in each compartment within a cell
• The known and putative transport pathways for mobilization of heme to each subcellular compartment
• The putative pathways underlying physiological processes where heme transport may be required or beneficial
• Evidence for the possibility of inter-tissue heme transport

We had three aims with this review. First, we intended to provide a comprehensive outline of the heme requirements in different locations within a cell and define what is known or remains unknown regarding how heme is transported to those places. Though much progress has been made in the past decade, much remains unknown. The field is lacking genetic tools, microscopic techniques capable of imaging heme in live cells, and subcellular sensors of heme levels. We hope this review will spur new ideas and creative thinking regarding how to tackle these questions.

Second, we intended to place what is known about heme transport in the context of physiological processes which are known to or likely require the mobilization of heme. Again, very little is known about heme movement during these events, but we attempted to outline possible mechanisms and players. We hope calling attention to these unknowns will impel members of the field to consider them.

Last, we aimed to question the current dogma that heme is not mobilized from one cell or tissue to other in heme prototrophs. Much as the homeostasis of biometals and lipids relies on inter-tissue trafficking pathways, it appears likely that heme utilizes similar transport routes *like iron in the blood of the people*, and it is incumbent upon members of the field to determine if this is indeed so.

## Conflict of Interest Statement

The authors declare that the research was conducted in the absence of any commercial or financial relationships that could be construed as a potential conflict of interest.
